# Investigation of Chromosomal Abnormalities and Microdeletion/ Microduplication(s) in Fifty Iranian Patients with Multiple Congenital Anomalies

**DOI:** 10.22074/cellj.2019.6053

**Published:** 2019-06-15

**Authors:** Akbar Mohammadzadeh, Susan Akbaroghli, Ehsan Aghaei-Moghadam, Nejat Mahdieh, Reza Shervin Badv, Payman Jamali, Roxana Kariminejad, Zahra Chavoshzadeh, Saghar Ghasemi Firouzabadi, Roxana Mansour Ghanaie, Ahoura Nozari, Sussan Banihashemi, Fatemeh Hadipour, Zahra Hadipour, Ariana Kariminejad, Hossein Najmabadi, Yousef Shafeghati, Farkhondeh Behjati

**Affiliations:** 1 Genetics Research Center, University of Social Welfare and Rehabilitation Sciences, Tehran, Iran; 2 Pediatric Neurology Research Center, Mofid Children’s Hospital, Faculty of Medicine, Shahid Beheshti University of Medical Sciences, Tehran, Iran; 3 Clinical Genetics Division, Mofid Children’s Hospital, Faculty of Medicine, Shahid Beheshti University of Medical Sciences, Tehran, Iran; 4 Children’s Medical Center, Pediatrics Center of Excellence, Tehran University of Medical Sciences, Tehran, Iran; 5 Cardiogenetic Research Laboratory, Rajaie Cardiovascular Medical and Research Center, Iran University of Medical Sciences, Tehran, Iran; 6 Genetic Counseling Center, Shahroud Welfare Organization, Shahroud, Iran; 7 Kariminejad-Najmabadi Pathology and Genetics Center, Tehran, Iran; 8 Department of Immunology and Allergy, Mofid Children’s Hospital, Faculty of Medicine, Shahid Beheshti University of Medical Sciences, Tehran, Iran; 9 Pediatric Infections Research Center, Research Institute for Children’s Health, Faculty of Medicine, Shahid Beheshti University of Medical Sciences, Tehran, Iran; 10 Sarem Fertility and Infertility Research Center (SAFIR), Sarem Women’s Hospital, Tehran, Iran; 11 Sarem Cell Research Center (SCRC), Sarem Women’s Hospital, Tehran, Iran

**Keywords:** Array Comparative Genomic Hybridization, Chromosomal Abnormalities, Congenital Abnormalities, Microdeletions, Multiplex Ligation-Dependent Probe Amplification

## Abstract

**Objective:**

Major birth defects are inborn structural or functional anomalies with long-term disability and adverse impacts on individuals, families, health-care systems, and societies. Approximately 20% of birth defects are due to chromosomal and genetic conditions. Inspired by the fact that neonatal deaths are caused by birth defects in about 20 and 10% of cases in Iran and worldwide respectively, we conducted the present study to unravel the role of chromosome abnormalities, including microdeletion/microduplication(s), in multiple congenital abnormalities in a number of Iranian patients.

**Materials and Methods:**

In this descriptive cross-sectional study, 50 sporadic patients with Multiple Congenital Anomalies (MCA) were selected. The techniques employed included conventional karyotyping, fluorescence in situ hybridization (FISH), multiplex ligation-dependent probe amplification (MLPA), and array comparative genomic hybridisation (array-CGH), according to the clinical diagnosis for each patient.

**Results:**

Chromosomal abnormalities and microdeletion/microduplication(s) were observed in eight out of fifty patients (16%). The abnormalities proved to result from the imbalances in chromosomes 1, 3, 12, and 18 in four of the patients. However, the other four patients were diagnosed to suffer from the known microdeletions of 22q11.21, 16p13.3, 5q35.3, and 7q11.23.

**Conclusion:**

In the present study, we report a patient with 46,XY, der(18)[12]/46,XY, der(18), +mar[8] dn presented with MCA associated with hypogammaglobulinemia. Given the patient’s seemingly rare and highly complex chromosomal abnormality and the lack of any concise mechanism presented in the literature to justify the case, we hereby propose a novel mechanism for the formation of both derivative and ring chromosome 18. In addition, we introduce a new 12q abnormality and a novel association of an Xp22.33 duplication with 1q43q44 deletion syndrome. The phenotype analysis of the patients with chromosome abnormality would be beneficial for further phenotype-genotype correlation studies.

## Introduction

Major birth defects are considered to be inborn structural or functional abnormalities which could be diagnosed prenatally, at birth, or later in infancy, or even in adulthood. Their consequence will be a long-term disability with major adverse effects on individuals, families, health-care systems, and societies (1). Approximately 20% and more than 10% of all neonatal deaths in Iran and worldwide (2) are caused by birth defects, respectively. The incidence of major birth defects in neonatals, resulting from genetic or partially genetic factors, is about 7.9 million per year (3). A fifth (20.2%) of the cases with birth defects are attributed to a known etiology comprising of chromosomal (15.8%) and genetic (3.8%) conditions. Teratogens are the cause of 0.8% of cases, 0.3% of them are caused by twinning, and four-fifths (79.8%) are categorized to be of unknown etiology (1). Nearly, 20 to 30% of the infants suffering from birth defects have multiple congenital anomalies (MCA) involving different organs. In cases where two or more major congenital abnormalities occur in several organs and the defects do not represent a sequence or a complex series, the case is classified as MCA (4).

Genetic factors are regarded as one of the most prominent etiologies of MCA (1). Also, chromosomal abnormalities have been known to be one of the leading causes of intellectual disability (ID) as well as congenital malformations. A large number of chromosomal defects can be detected immediately through high-resolution karyotyping technique. But, the resolution of the cytogenetic analysis is restricted to around 5 to 10 Mb. Among molecular cytogenetic techniques, fluorescence in situ hybridization (FISH) and multiplex ligation-dependent probe amplification (MLPA) are two of the targeted tests used to detect submicroscopic chromosome abnormalities. Genomewide molecular cytogenetic tests like array comparative genomic hybridisation (array-CGH) can identify a variety of copy number variants (CNVs) associated with MCA. Array-CGH, as a powerful test, is applied to investigate individuals with MCA, ID, and autism spectrum disorders (5). In the current study, we aimed at identifying the chromosomal abnormalities and microdeletion/microduplication(s) in 50 Iranian patients with MCA. The patients manifested diverse phenotypes including ID/developmental delay (DD), and at least one major congenital anomaly in another organ. Existence of additional minor dysmorphic features was also considered among the factors representing chromosomal abnormalities which led to an increase in the detection rate.

## Materials and Methods

### Selection of patients

In this descriptive cross-sectional study conducted during the three past years from April 2015 to May 2018, clinical evaluation was performed for 50 selected sporadic patients suffering from MCA preferably born to unrelated parents and referred to us by experienced clinical specialists for genetic investigation from all over the country. All of the patients had ID/DD with at least one major anomaly and additional minor dysmorphic features. All of the steps taken for testing and procedures were fully explained for all of the probands’ parents, and signed informed consent forms from all of the participants were obtained for publishing any information or accompanying photographs. This study was approved by the Ethics Committee of the University of Social Welfare and Rehabilitation Sciences (IR.USWR.REC.1394.186).

### Conventional karyotyping

A conventional cytogenetic study was carried out on peripheral blood lymphocytes using GTG high-resolution banding technique according to standard protocols for all patients (6). Twenty GTG banded metaphases were examined through the complete analysis of each individual sample. Chromosome analysis was performed according to ISCN 2016 (7).

### Fluorescence in situ hybridization

Metaphase FISH was done only for patient 9 using the centromeric probe of chromosome 18 (Kreatech/ Leica Biosystems Buffalo Grove, IL, USA, <uri>http://www. leicabiosystems.com</uri>) according to Kreatech protocol (8). Two hundred cells were examined to characterize the ring chromosome origin defined in 37% of cells by highresolution GTG banding.

### Multiplex ligation-dependent probe amplification

MLPA technique was done using P245 Microdeletion Syndromes-1 kit (MRC-Holland, Amsterdam, the Netherlands) for the patients suspected of microdeletion/ microduplication syndromes. MLPA protocol was performed based on the instructions provided by the manufacturer, MRC Holland, and the data were analyzed utilizing capillary electrophoresis in the 3130XL DNA Analyzer, Coffalyser. Net software (MRC-Holland, Amsterdam, the Netherlands) and Gene Marker software version 2.7.0 (Softgenetics, State College, PA, USA). Abnormal results obtained with MLPA assay were repeated and if different, further investigations were carried out with array-CGH. MLPA studies were done for the patients’ parents with abnormal results in order to determine the causal role (de novo or inherited) of microdeletions.

### Array comparative genomic hybridization

The analysis of oligonucleotide array-CGH for Genomic DNA was carried out. To do so, the BlueGnome CytoChip ISCA 8×60 K v2.0 wholegenome oligo array was utilized for patients 1, 5, 9, 13, and 41. This array included intragenic and intergenic probe spacings of about 48 kb and 70 kb, respectively and high probe density in 500 clinically important regions. CytoSNP-850K v1.1 BeadChip overall effective resolution of about 18 Kb was used for patient 27. The tests were performed based on the manufacturer’s protocol. INNOPSYS 910 laser scanner was utilized for scanning according to the recommended protocol of the manufacturer. Image analysis and base calling were carried out employing the BlueFuse Multi-version 3 analysis software for oligo array and Multi-version 4.4 analysis software for SNP array. The analysis of the samples was carried out two times against two non-identical controls and representation of minimum three clones on the platform in both of the experiments was considered to approve the imbalances. To evaluate the called CNVs, we utilized the standards and guidelines of American College of Medical Genetics and Genomics to interpret the postnatal constitutional CNVs (5). All chromosome coordinates are on the basis of GRCh37. p13/hg19.

### Results

Based on the selection criteria, the patients had MCA, i.e., having ID/DD and at least one major congenital anomaly in another organ plus additional minor facial dysmorphic features. The clinical characteristic features of all patients with MCA are summarized in Table 1. The participants included 25 male and 25 female patients. Their ages ranged from 7 months to 21 years with a median age of 3.25 years and an average of about 5.1 years (Table 1).

Excluding ID/DD, the most common clinical features from the highest frequent to the lowest frequent were congenital heart defects in 45 patients (90%), craniofacial dysmorphic features in 31 patients (62%), musculoskeletal, ear and genitourinary abnormalities, each one in 19 patients (38%), eye abnormalities in 16 patients (32%), gastrointestinal abnormalities in 5 patients (10%), and endocrine abnormalities in 3 patients (6%) (Table 2).

**Fig.1:**
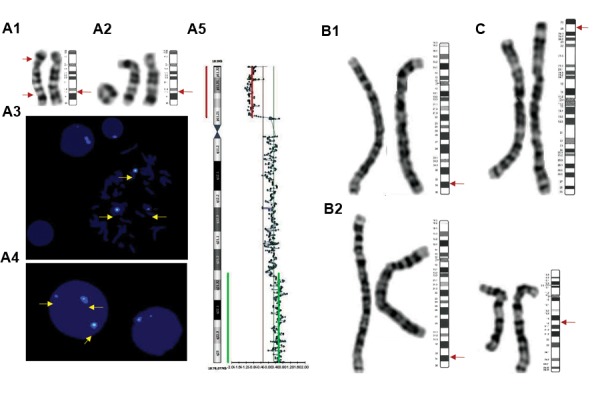
Results of cytogenetic/molecular cytogenetic analysis of patients 9, 17, and 41. A1. Abnormal G-banded chromosomes 18 observed in all spreads, A2. 37% of spreads included ring 18, der(18) and normal 18 from left to right, A3, A4. FISH results using centromeric probe for chromosome 18 (arrowed), A5. Array-CGH profile of chromosome 18 utilizing the BlueGnome CytoChip ISCA 8x60K v2.0 whole genome oligo array, B1. G-banded der(4), B2. Balanced translocation of chromosomes 4 and 12, and C. G-banded chromosomes 3 with 3p25.3p26.3 deletion. The normal idiograms of the mentioned chromosomes are in accordance with ISCN 2016 (7).

In patient 9, a 31-month-old boy from consanguineous parents (half first cousins) and born to a 27-year-old G1P1L1A0 (G: gravid, P: parity, L: live birth, A: abortus) mother, the clinical features were global DD, PDA, PFO, optic nerve hypoplasia, hypogammaglobulinemia, facial dysmorphic features, microcephaly, and mild ventriculomegaly in frontal horns in the axial spiral CT-scan of the brain without contrast, and benign extra-axial hydrocephalus and atrophic changes in the brain magnetic resonance imaging (MRI). The karyotype analysis by highresolution GTG banding showed 46,XY, der(18) [12]/46,XY, der(18), +mar[8] dn (Fig .1A1, 2). Since in both cell lines of the patient the short arm of one chromosome 18 (18p) was abnormal and one cell line had an additional ring chromosome, one hundred cells were screened to obtain the percentage of the cells with marker ring chromosome. The karyotype analyses of the proband’s parents were normal. Metaphase FISH using the centromeric probe for chromosome 18 indicated three hybridization signals for chromosome 18 in 37% of the cells scored which was consistent with mosaic trisomy 18, nuc ish(D18Z1×3)[37]/(D18Z1×2) [63] dn (Fig .1A3, 4). Further investigations using array-CGH demonstrated the exact breakpoints of the derivative chromosome 18 and its deletion and duplication (Fig .1A5).

**Table 1:** The clinical features of 50 patients with MCA


Patient	Sex	Age	Clinical findings

1	F	14 month	DD, SVAS, SVPS, IUGR, Hypotonia, Left visual defect, Facial dysmorphic features, Strabismus, ADHD, Dental problems, Congenital hypothyroidism
2	M	25 month	DD, VSD, ASD, Pulmonary hypertension, Microcephaly, Facial dysmorphic features, UDT, Small testes
3	F	8 Y	ID, TOF, PDA, Microcephaly, DD, Aggressiveness, Umbilical hernia, Flat feet, Syndactyly
4	M	3.5 Y	DD, ASD, PDA, Bilateral congenital glaucoma, Hypotonia, Bilateral club feet, Bilateral hip dislocation (grade 4), Bilateral hydronephrosis, Bilateral inguinal hernia and hydrocele, Hypothyroidism
5	F	5.5 Y	ID, Large ASD, Epilepsy, Autistic behavior, Macrocephaly, Dolicocephaly, Facial dysmorphic features, Sparse hair, Joint laxity, Severe left renal reflux
6	F	6 Y	ID, VSD, ASD, Microcephaly, Bilateral club feet, Joint stiffness
7	M	2.5 Y	DD, Large ASD, Bilateral SNHL, Bilateral hydronephrosis, Dysphagia, Hypotonia, Polydactyly
8	M	3.5 Y	DD, TR, Facial paralysis, Left SNHL
9	M	31 month	Global DD, PDA, PFO, Hypotonia, Optic nerve hypoplasia, Facial dysmorphic features, Recurrent lower respiratory tract infection, Hypogammaglobulinemia, Dysphagia, Microcephaly, Speech disorder, Hypertelorism, Micrognathia, Clinodactyly, Rocker bottom feet, Short stature, Fecal/urinary incontinence
10	M	4 Y	DD, Severe AS, Severe PS, ASD, Facial dysmorphic features, Brachydactyly, Clinodactyly, Flat feet, Hirsutism
11	F	4 Y	DD, TOF, IUGR, Hypotonia, ADHD, Insomnia, Microcephaly, Facial dysmorphic features, Brachydactyly, Bilateral simian creases, Low-set ears
12	M	1.5 Y	DD, TOF, Hypotonia, Facial dysmorphic features, Low-set ears, Bilateral UDT
13	M	3 Y	DD, VSD, AR, Bilateral SNHL, Facial dysmorphic features, Bilateral UDT, Speech delay, Malformed, Posteriorly rotated and low set ears, Simian crease, 5^t^^h^ toe clinodactyly, Umbilical hernia, Sacral mongolian spot, Right lower limb paresis, Joint contractures, Bilateral pachygyria
14	M	4.5 Y	DD, TOF, Hypotonia, Bilateral SNHL, Urinary reflux
15	M	3 Y	DD, Large ASD, Hypotonia, Microcephaly, Facial dysmorphic features, High-arched palate
16	F	3 Y	DD, ASD, Hypotonia, Facial dysmorphic features, Speech delay, Macrocephaly, Wide anterior fontanel
17	M	8.5 month	ID, DD, PDA, ASD, Hypotonia, Facial dysmorphic features, Infantile spasms and myoclonic jerks, Hearing loss, Bilateral simian creases, Corpus callosum hypoplasia, Growth retardation, Abnormal EEG, Microcephaly, Short neck, Malformed ears, Low-set ears, High-arched palate, Camptodactyly, Joint hyperlaxity, Imperforated anus, Umbilical hernia, Bilateral UDT, Small kidneys
18	M	5.5 Y	ID, Large ASD, PS, PDA, Hypotonia, Long philtrum, Visual defect, Bilateral inguinal hernia
19	F	3.5 Y	DD, Severe PS, Facial dysmorphic features
20	F	3 Y	DD, TOF, Bilateral congenital anophthalmia, Absence of manubrium
21	F	2 Y	DD, ASD, Bilateral SNHL, Facial dysmorphic features, Right periauricular and periorbital tags, Rectovaginal fistula, Right lower lid coloboma
22	M	7 Y	ID, VSD, Hypotonia, Corpus callosum hypoplasia, DD, Facial dysmorphic features
23	M	2.5 Y	DD, TGA, Bilateral SNHL, Strabismus, High-arched palate, Clinodactyly
24	M	2 Y	DD, Large VSD, PDA, Pulmonary hypertension, Left SNHL, Microcephaly, Bilateral UDT, Ambiguous genitalia
25	F	5.5 Y	ID, Large VSD, Pulmonary hypertension, Hypotonia, Visual defect, Epilepsy, Bilateral simian creases, Microcephaly, Hyperreflexia
26	M	2 Y	DD, TOF, Hypotonia, Facial dysmorphic features
27	M	18 Y	ID, TOF, DD, Microcephaly, Hypotonia, Severe scoliosis, Short stature, Facial dysmorphic features, Myopia, Strabismus, High-arched palate, Low-set ears, Left UDT, Growth retardation, Speech delay, Flat feet
28	M	2.5 Y	DD, Large ASD, TA, Short and bifid sternum, Hypotonia
29	M	2 Y	DD, Large VSD, PS, Dextrocardia
30	M	3 Y	DD, PDA, PS, PFO, Hypotonia, Bilateral ptosis, Strabismus, Bilateral UDT
31	F	10 month	DD, TA, PDA, VSD, ASD, Dolicocephaly
32	M	15.5 Y	ID, VSD, Epilepsy, Autism, Facial dysmorphic features, Retrognathia, Low-set ears, Joints hypermobility, Bilateral club feet, Polydactyly (right foot)
33	F	1.5 Y	DD, ASD, Bilateral SNHL, Cleft palate, Facial dysmorphic features, Hip dislocation, Simple cyst (Left kidney)
34	F	8 Y	ID, ASD, Autism, Hirschsprung, Bilateral inguinal hernia, Hypopigmentation of neck and back
35	F	7 month	DD, ASD, Peripheral PS, Ascending aorta dilatation, Bilateral cataracts, Facial dysmorphic features, Left ptosis, Low set ears, Micrognathia
36	M	2 Y	DD, PFO, Moderate bilateral SNHL, Hypotonia, Convulsion, Abnormal EEG, Hydrocephalus, Bilateral optic nerve atrophy
37	F	1 Y	DD, Large VSD, PFO, Pulmonary hypertension, Macroglossia, Facial dysmorphic features, Flat feet
38	F	12 Y	ID, TR, MVP, Macrocephaly, Ventriculomegaly (in MRI), Short stature, FTT, Ureteral stenosis, Facial dysmorphic features, Left ptosis, Photophobia, Ichthyosis, Sparse hair
39	F	15 month	DD, Dilated right atrium and ventricle, Dilated pulmonary artery, Hypotonia, Chest deformity, Vermis hypoplasia, Prominent diameters frontal horns of lateral ventricles in brain sonography
40	F	20 month	DD, ASD, PFO, Choanal atresia, Bilateral lower lid coloboma, Low-set ears, Brain hemiatrophy, Macroglossia
41	F	10 Y	ID, Autism, Facial dysmorphic features, Microcephaly, Bilateral SNHL, Epilepsy, Polydactyly (4 limbs), Low birth weight, Growth retardation, Abnormal EEG, Hypotonia, Trigonocephaly, Short stature, Triangular face, Ptosis, Low-set ears, Posteriorly rotated ears, Retrognathia, Micrognathia, Downturned mouth, High-arched palate, Fecal/urinary incontinence, Feeding problems
42	F	15 month	DD, VSD, ASD, RVH, Choanal atresia, Strabismus, Nasolacrimal duct obstruction, Bulbous nose
43	F	21 Y	ID, Autism, ADHD, Insomnia, Severe obesity, Aggressiveness
44	F	7 Y	ID, Autism, Bilateral lower lid coloboma, Facial dysmorphic features, Microcephaly, Retrognathia
45	F	11 Y	ID, Autism, Diabetes mellitus
46	F	10 Y	ID, Autism, Facial dysmorphic features, Low-set ears, Urinary incontinence, Right small kidney
47	F	7.5 Y	ID, VSD, Epilepsy, Postaxial polydactyly (4 limbs), Syndactyly
48	M	8.5 Y	ID, VSD, ASD, Facial dysmorphic features, Cleft lip and palate, Strabismus
49	M	6.5 Y	ID, VSD, DD, Facial dysmorphic features, Left ureteral stenosis
50	M	9 Y	ID, VSD, Epilepsy, Bilateral SNHL, Facial asymmetry, Strabismus, Microcephaly, Cleft lip/palate


MCA; Multiple congenital anomalies, F; Female, M; Male, AR; Aortic regurgitation, AS; Aortic stenosis, ASD; Atrial septal defect, DD; Developmental delay, FTT; Failure to thrive, ID; Intellectual disability, IUGR; Intrauterine growth restriction, MVP; Mitral valve prolapse, PDA; Patent ductus arteriosus, PFO; Patent foramen ovale, PS; Pulmonic stenosis, RVH; Right ventricular hypertrophy, SNHL; Sensorineural hearing loss, SVAS; Supravalvular aortic stenosis, SVPS; Supravalvular pulmonic stenosis, TA; Tricuspid atresia, TGA; Transposition of the great arteries, TOF; Tetralogy of fallot, UDT; Undescended testis/ testes, VSD; Ventricular septal defect, ADHD; Attention deficit/hyperactivity disorder, EEG; Electroencephalography, TR; Tricuspid regurgitation, and MRI; Magnetic resonance imaging.

**Table 2:** Frequency of main phenotypic manifestations of 50 patients with MCA categorized by organ systems


Phenotype		n (%)

Craniofacial		31 (62)^*^
	Facial dysmorphic features	27 (54)
	Microcephaly	13 (26)
	Macrocephaly	3 (6)
	Cleft lip/palate	3 (6)
	Dolicocephaly	2 (4)
Central nervous system		50 (100)
	ID/DD	50 (100)
	Hypotonia	20 (40)
	Epilepsy	8 (16)
	Corpus callosum agenesis	2 (4)
Cardiovascular system		45 (90)^*^
	ASD	19 (38)
	VSD	15 (30)
	PS	8 (16)
	PDA	8 (16)
	TOF	7 (14)
	Pulmonary hypertension	4 (8)
	PFO	4 (8)
	AS	2 (4)
Musculoskeletal system		19 (38)^*^
	Short stature	4 (8)
	Polydactyly	4 (8)
	Club foot	3 (6)
	Syndactyly	2 (4)
	Brachydactyly	2 (4)
	Clinodactyly	2 (4)
Ear		19 (38)^*^
	SNHL	11 (22)
	Low-set ear (s)	9 (18)
Eye		16 (32)^*^
	Strabismus	6 (12)
	Hypertelorism	5 (10)
	Ptosis	4 (8)
	Visual defect	3 (6)
	Lower lid coloboma	3 (6)
	Optic nerve hypoplasia	2 (4)
	Glaucoma	1 (2)
	Cataract	1 (2)
Genitourinary system		19 (38)
	UDT	7 (14)
	Urinary incontinence	3 (6)
	Small kidney	2 (4)
	Urinary reflux	2 (4)
	Small testes	1 (2)
	Hydrocele	1 (2)
	Ureteral stenosis	1 (2)
	Rectovaginal fistula	1 (2)
	Simple kidney cyst	1 (2)
Gastrointestinal system		5 (10)
	Dysphagia	2 (4)
	Fecal incontinence	2 (4)
	Hirschsprung	1 (2)
Endocrine system		3 (6)
	Hypothyroidism	2 (4)
	Diabetes mellitus	1 (2)
Miscellaneous		
	Autism	8 (16)
	ADHD	3 (6)
	Growth retardation	3 (6)
	Choanal atresia	2 (4)
	Hypogammaglobulinemia	1 (2)


*; More than one abnormality may be observed in one patient, MCA; Multiple congenital anomalies, ID; Intellectual disability, DD; Developmental delay, ASD; Atrial septal defect, VSD; Ventricular septal defect, PS; Pulmonic stenosis, PDA; Patent ductus arteriosus, TOF; Tetralogy of fallot, PFO; Patent foramen ovale, AS; Aortic stenosis, SNHL; Sensorineural hearing loss, UDT; Undescended testis/testes, and ADHD; Attention deficit/hyperactivity disorder.

The clinical findings for patient 9 were compared with the previously reported patients in the literature and the ECARUCA database (http://www.ecaruca.net) (9) in Table 3.

The cytogenetic and molecular cytogenetic results of 8 patients were reported in detail in Table 4.

**Table 3:** The clinical features of patient 9 compared to those previously reported with 18p deletion, 18q duplication, mosaic ring(18), and full trisomy


Signs and symptoms	18p11.1-pter del (case ID 4621) (9)	18p11.21 p11.32 del (case ID 4772) (9)	18p11.21-pter del (10)	18p deletion (11)	18q21.3- q23 dup (12)	18q21.3- q23 dup (12)	Mosaic ring18 (13)	Mosaic ring18 (14)	Full trisomy 18 (15)	18p11.21p11.32 del and 18q21.31q23 dup (patient 9)

Growth retardation									+	+
ID/DD	+	+		+	+	+	+	+	+	+
Microcephaly	+								+	+
Defects of CNS									+	+
Speech disorder				+			+	+		+
Hypotonia	+		+		-		+	+	+	+
Heart defects						-	-		+	+
Optic nerve hypoplasia					-					+
Ptosis of eyelids	+	+		-			+		+	-
Strabismus			+	+	+		+			-
Hypertelorism			+	-	-		+		+	+
Down/up slanting-palpebral fissures		+	-		-	-	+		+	+
Low-set ears		+	+		-	-	+	+	+	+
Wide nasal bridge	+									+
Long/short philtrum					-		-	+	+	+
High-arched palate			-	+	-	+		+	+	+
Micrognathia			-		-	+	+		+	+
Clinodactyly			+		-		+	+	+	+
Rocker bottom feet									+	+
Short stature	+							+	+	+
IgA deficiency			-	+	-		+			+
IgG deficiency				-	-		+			+
IgM deficiency				-	-		+			+
Feeding problem	+									+
Genital malformations			-		-	-	-		+	+
Hernia		+			-	+	-			+


ID; Intellectual disability, DD; Developmental delay, CNS; Central nervous system, +; Indicates presence, -; Indicates absence, and blank space; Indicates not available/not reported data.

**Table 4:** Characterization of chromosomal abnormalities detected in 8 patients with MCA


Patient	Cytogenetic band	Chromosomal sequence	Size	Del/Dup	Significant genes	Known syndromes	Inheritance

1	7q11.23	72,766,343-74,133,303	1.37 Mb	Del	22 OMIM genes ELN, LIMK1, RFC2, FKBP6, FZD9, STX1A, GTF2IRD1, BAZ1B	Williams-Beuren syndrome	De novo
5	5q35.2q35.3	175,559,373- 177,422,731	1.86 Mb	Del	25 OMIM genes includingNSD1	Sotos syndrome	De novo
9	18p11.21p11.32	148,992-13,448,995	13.3 Mb	Del	44 OMIM genesTGIF1, LIPIN 2, LAMA1	-	De novo
	18q21.31q23	54,532,626-78,012,800	23.5 Mb	Dup	60 OMIM genes includingMALT1, PIGN	-	
	18p11.21q21.31	(Mosaic ring, 37% of cells)	-	Dup	-	-	
13	22q11.21	18,706,023-21,561,492	2.86 Mb	Del	41 OMIM genes including TBX1	Digeorgesyndrome	De novo
17	12q15-qter	-	-	Dup	HAND2	-	Maternal
	4q33	-	-	Del	-	-	
27	1q43q44	242,003,539-249,218,992	7.2 Mb	Del	19 OMIM genes including AKT3, NLRP3, HNRNPU, SMYD3 KIF26B, ZBTB18	Mental retardation- autosomal dominant 22 (MRD22)	De novo
	Xp22.33	60,814-601,612	541 Kb	Dup	4 OMIM genes including SHOX, PPP2R3B, PLCXD1, GTPBP6	-	
35	16p13.3	-	-	Del	CREBBP	Rubinstein-Taybi syndrome 1	De novo
41	3p26.3p25.3	93949_11504861	11.4 Mb	Del	SETD5, BRPF1, CHL1, CNTN4, SLC6A1, SLC6A11	3p deletion syndrome	De novo or Paternal
	10q26.3	135243049-135372492	130 Kb	Dup	CYP2E1, SYCE1	-	


MCA; Multiple congenital anomalies, Del; Deletion, and Dup; Duplication.

Patient 17, an 8.5-month-old boy born to nonconsanguineous parents with DD, infantile spasms and myoclonic jerks, abnormal EEG, PDA, ASD, Facial dysmorphic features, Hypotonia, and Corpus callosum hypoplasia (Table 1), showed an additional segment of 12q15-qter origin on 4q33 in karyotype analysis. The patient father’s karyotype was normal, but his mother’s karyotype showed a reciprocal balanced translocation between long arms of chromosomes 4 and 12 46,XX,t(4;12)(q33;q15). The clinical features of patient 17 were compared with those of the previously reported patients who had overlapping genotypes with patient 17. The information was obtained from the ECARUCA (9) and DECIPHER (16) databases (Table 5).

**Table 5:** The clinical features of patient 17 compared to the cases previously reported with partial 12q duplications and those with partial deletions of 4q


Signs and symptoms	12q21.31 - 12q21.32 dup patient ID 264283 (16)	12q21.32 - 12q23.1 dup patient ID 258582 (16)	12q23.3- q24.31 dup case ID 4588 (9)	12q24.1- q24.3 dup case ID 4053 (9)	4q33-qter del (17)	4q33-qter del (18)	4q33-qter del (18)	4q33-qter del (19)	12q15-qter dup and 4q33-qter del (patient 17)

Sex	M	F	M	F	M	F	M	M	M
Growth retardation			+		+			-	+
ID	+	+	+Severe		+	-	+	+	+
Seizures/Abnormal EEG		+					+	-	+
Microcephaly	+		+	+	+	+			+
Hypoplasia/agenesis of corpus callosum			+	+					+
Frontal bossing/high forehead									+
Hypotonia		+	+					-	+
Heart defects					-		+	+	+
Short neck			+	+	-			+	+
Hypertelorism			+		+			-	+
Epicanthal folds			+	+		+		-	+
Slanting palpebral fissures				Up		Up	Up	Up	Down
Malformed ears			+	+	-	+	+	+	+
Low-set ears			+	+			+	-	+
Micrognathia			+	+	+	+	+	-	-
Flat malar chin/flat mid-face							+	+	+
Thin upper/lower lip			+	+				+	-
High-arched palate		+						-	+
Down-turned corners of the mouth			+	+		+		+	-
Abnormality of the teeth		+	+					+	-
Abnormal palmar creases			+	+	-			+	+
Camptodactyly					-	+		-	+
Prominent/Bulbous nasal tip						+		-	+
Anteverted nares			+		+	+	+	+	+
Depressed/flat nasal bridge					+	+	-	-	+
High/prominent nasal bridge			+	+			+	-	-
Wide nasal bridge				+				+	+
Short stature			+	+	+	+		-	-
Foot deformity				+	+	+	+	+	+
Sacral dimple/sinus			+	+	+	+		-	+
Imperforated anus								-	+
Hearing loss								-	+Bilateral
Umbilical hernia									+
Cryptorchidism			+		+			-	+
Small kidneys									+


EEG; Electroencephalography, F; Female, ID; Intellectual disability, M; Male, +; Presence, -; Absence, and blank spaces indicate not available/not reported data.

Patient 41, a 10-year-old boy born to non-consanguineous healthy young parents with ID, autistic behavior, impaired social functioning, facial dysmorphic features, language delay, and microcephaly, bilateral SNHL, polydactyly (4 limbs), epilepsy, abnormal EEG, hypotonia, and trigonocephaly (Table 1), carried a terminal deletion of 3p in conventional cytogenetic characterization: 46, XY,del(3)(p25.3p26.3) (Fig .1C). The karyotype analysis of the proband’s mother showed an apparently normal female karyotype, but the father’s blood sample was unavailable. Further investigations using array-CGH confirmed 11.4 Mb terminal deletion of the short arm of chromosome 3 and about 130 Kb terminal duplication of the long arm of chromosome 10. Given the terminal 10q duplication, the probability that the 3p26.3p25.3 deletion was inherited from his father with 46,XX,t(3;10) (p26.3p25.3; q26.3) is high.

The karyotype of patient 27, an 18-year-old boy from non-consanguineous parents and born to a 31-year-old G7P6L6A1 mother at term, had been reported as a normal male in his medical documents. The result of further investigation using P245 Microdeletion Syndromes-1 kit was negative for the microdeletion/microduplication syndromes. The clinical features were ID, tetralogy of fallot (TOF), microcephaly, severe scoliosis, short stature, speech delay, and facial dysmorphic features (Table 1). In the conventional cytogenetic investigation repeated, a suspicion for terminal 1q deletion was observed. Array- CGH was performed and reported two inherited CNVs: A 7.2 Mb deletion of the terminal end of 1q43q44 and a 541 Kb duplication of Xp22.33. The array-CGH analyses performed for both of the parents were normal.

Patient 1 was a 14-month-old girl from healthy and young non-consanguineous parents born through normal vaginal delivery to a G1P1L1A0 mother at term. The clinical characteristics were ID, intrauterine growth restriction (IUGR), hypotonia, hypothyroidism, supravalvular aortic stenosis, supravalvular pulmonary stenosis, left visual defect, attention deficit/hyperactivity disorder, strabismus, long philtrum, and thick lips. Conventional cytogenetic analysis was normal. MLPA screening demonstrated a deletion of 7q11.23 consistent with Williams-Beuren syndrome. Further assays using array-CGH confirmed about 1.37 Mb deletion including 22 OMIM genes. Array-CGH analyses of the parents were normal.

Patient 5 was a 5.5-year-old girl, born through normal vaginal delivery to healthy and young non-consanguineous parents following a full-term pregnancy. Her mother was G2P1L1A1. She had suffered constipation for the first six months after birth. At the age of 14 months, the renal cortical scan revealed lesser activity in the lower pole of the left kidney because of pyelonephritis. During voiding cystourethrogram, severe urinary tract reflux was present in the left kidney. She also had a large ASD, ID, seizure attacks, autistic behavior, macrocephaly, dolicocephaly, frontal bossing, fish-shaped mouth, sparse hair, and joint laxity. She can say up to 5 words. The karyotype analysis was normal. MLPA assay revealed a deletion of 5q35.3 consistent with Sotos syndrome. Further investigations using array-CGH confirmed 1.86 Mb deletion including 25 OMIM genes. Array-CGH analyses of the parents were normal.

Patient 13 was a 3-year-old boy who was the product of the first pregnancy and cesarean delivery of nonconsanguineous parents. The clinical features were widow’s pick hair, down-slanting palpebral fissures, long eyelashes, deep set eyes, malformed, posteriorly rotated and low set ears, upward nose and nares, long philtrum, small mouth, high arched palate, high pitched cry, wheezing respiratory sounds, cardiac surgery scar, simian crease, 5th toe clinodactyly, umbilical hernia, sacral mongolian spot, overriding of 1st toe with 2nd toe, right lower limb paresis, joint contractures, right sided tippy-toed walking and bilateral undescended testes. Axial spiral CT-scan of the brain without contrast media showed bilateral pachygyria at frontotemporoparietal lobes due to abnormal neuronal migration. The karyotype analysis was normal. MLPA assay detected a deletion of 22q11.21 consistent with DiGeorge syndrome. Further investigations using array- CGH confirmed 2.86 Mb deletion including 41 OMIM genes, arr 22q11.21 (18,706,023-21,561,492) x1 dn. Array-CGH analyses of the parents were normal.

Patient 35 was a 7-month-old girl born through cesarean section to a healthy consanguineous (first cousin) couple. Her mother was G1P1L1A0. She had bilateral cataracts, hypertelorism, left ptosis, frontal bossing, low set ears, long philtrum, broad nasal bridge, micrognathia, a borderline lactic acid and normal creatinine in urine, ascending aorta dilatation, peripheral pulmonary stenosis, and small ASD. Conventional cytogenetic analysis showed a normal female karyotype. The MLPA technique indicated a deletion of 16p13.3 consistent with Rubinstein-Taybi syndrome. The sample assay was carried out twice. The parents’ karyotype analyses and MLPA assays were normal.

## Discussion

Based on several studies, the diagnostic yields of conventional cytogenetic studies in patients with MCA, global DD, and autism spectrum disorders, barring the known trisomy syndromes, have been reported to be about 3% (20). In this study, we identified chromosomal abnormalities, microdeletions, and microduplications for 8 patients (16%) using conventional karyotyping technique [3 patients (6%)], MLPA method [4 patients (8%)], and array-CGH [1 patient (2%)]. In addition to the sample size limitations, the adoption of inflexible criteria for selecting patients and the design type of the study could have contributed to the raising the diagnostic rate of this study.

Well-defined microdeletion syndromes were the most common cause of MCA in this study, consistent with the results of previously reported studies (21). The clinical features of four patients 1, 5, 13, and 35 are congruent with known microdeletion syndromes namely Williams-Beuren syndrome, Sotos syndrome, DiGeorge syndrome, and Rubinstein-Taybi syndrome, respectively. Consequently, they are not discussed in detail.

According to a hypothesis, two critical regions are within the long arm of chromosome 18 (18q), including 18q12.1-q21.2 and 18q22.3-qter. Duplication occurrence is essential in both proximal and distal regions in order to cause the typical clinical features of a trisomy 18 to be manifested (12). In patient 9, the former duplication is considered as a mosaic trisomy and the latter as a full trisomy.

As a chromosomal abnormality, partial deletion of 18p is more common than partial duplication of 18q (22). Mostly, a balanced translocation or inversion carried by one parent is the leading cause of partial trisomy of 18q (15). The real mechanism of mosaic ring 18 formation in patient 9 is unknown. Although his parents’ karyotypes were normal, there was a recombinant pattern of pericentric inversion and an extra ring in the patient’s karyotype. The possible mechanism for the formation of both derivative and ring chromosome 18 could, most probably, have initiated with an inverted chromosome 18 (p11.21– q21.31), created during meiosis in the gamete of one parent (23). Postzygotic mitosis resulted in two partially q duplicated and partially p deleted recombinants (22). The genetic size of 18p is almost 16 Mb with a common breakpoint cluster located in the pericentromeric region with a length of 4 Mb. The partial deletion of 18p in this patient was inside the breakpoint cluster spanning 13.3 Mb. It should be noted that the 18q21.3 segment is one of the common fragile sites of chromosome 18 (24). One of the recombinants had seemed to have two breakpoints in 18q21.31 and 18p11.21 bands and the sticky ends of the segment between them fused and formed the ring chromosome 18. The broken segments were lost and some of the rings created were deleted in the next mitoses. The formation of ring chromosome 18 may be a novel escape mechanism in this patient with a pentasomy of 18q21.31- qter segment (25).

Most clinical phenotypes of patient 9 overlapped with the clinical manifestations of partial 18p deletion, partial 18q duplication, and mosaic ring chromosome 18 syndromes. The phenotypic variations could be explained based on the locations and the lengths of the duplicated or deleted segments with a mosaic triplicated segment between them considering the co-occurrence of different types of chromosome 18 abnormalities in this patient.

Litzman et al. (13) reported an agammaglobulinemic 14-year-old girl with a mosaic ring chromosome 18. Her karyotype turned out to be: 45, XX,-18[5]/46, XX, dic r(18)[6]/46, XX, r(18)[89]. IgA deficiency/absence with normal ranges of IgG and IgM has been previously reported in association with 18p deletion (11). To the best of our knowledge, the proband is the first patient to be reported with partial monosomy of 18p, partial 18q duplication, and an extra mosaic ring chromosome 18 associated with hypogammaglobulinemia.

Based on the information obtained, no patient has been previously reported with partial duplication of 12q with the breakpoint at 12q15 to qter. However, although chromosome 4q deletions occur rarely, 4q33-qter deletions have been reported several times (17). It seems that some clinical features of patient 17 like hypoplasia of corpus callosum, hypotonia, and high-arched palate are also observed in the cases with partial 12q duplication. In contrast, some of the other physical characteristics such as heart defects, flat midface, camptodactyly, and depressed nasal bridge are consistent with phenotypic features of terminal deletion of 4q33-qter. However, a number of major anomalies in patient 17 including bilateral hearing loss, imperforated anus, umbilical hernia, and small kidneys have not been reported in the patients with partial 12q duplication or partial 4q deletion. The large size of 12q15-qter duplication could be the cause of these extra phenotypes. Homo sapiens HAND2 gene located on 4q34.1 has been known as the responsible gene whose proteins play a crucial role in developing ventricular chambers, cardiac morphogenesis, forming right ventricle and aortic arch arteries as well as developing limb and branchial arch. The haploinsufficiency of this gene in 4q deletion may justify inborn heart anomalies (i.e., VSD and ASD), growth retardation, ID, digital anomaly (i.e., clinodactyly and oligodactyly), and craniofacial dysmorphism including a cleft palate (26).

Approximately 11.4 Mb deletion of the distal segment of 3p containing 46 OMIM genes has led to severe and different phenotypes in patient 41.

The distal deletion of 3p is a rare contiguous gene syndrome with the variable spectrum of major anomalies such as developmental delay, growth retardation, autism, hearing loss, renal anomalies, heart defects, and craniofacial dysmorphic presentations. Given common facial dysmorphic features and ID among patients with intragenic sequence variants and microdeletions affecting SETD5, it has been suggested that this gene plays a vital role in ID and also has a significant contribution to the core phenotype of 3p deletion syndrome (27).

It seems that ID severity in patient 41 is due to the haploinsufficiency association of *SETD5* and *BRPF1* genes. *CHL1* gene deletion associated with mild ID and learning difficulties (28) may also contribute to this severity. Furthermore, some clinical manifestations specially ptosis and short stature may occur because of *BRPF1* gene deletion (29).

The autistic behavior of patient 41 may be because of *CNTN4* (contactin 4), previously introduced as a strong candidate gene associated with autism spectrum disorders (30). Two genes namely *SLC6A1* and *SLC6A11* encoding GABA transporter proteins have been proposed as possible candidates for seizures/EEG abnormalities, ataxia, and ID (31).

1q43q44 deletion in patient 27 with the size of 7.2 Mb included 126 HGNC and 19 OMIM genes. Of these genes, *AKT3* gene haploinsufficiency has been linked with microcephaly in previously reported cases with 1q43q44 deletion syndrome (32). However, because of the presentation of microcephaly in the patients with intact *AKT3* gene, the other candidate genes such as *NLRP3*, *HNRNPU, SMYD3,* and *KIF26B* have been suggested to cause microcephaly (33). Nonetheless, *AKT3* deletion may explain the severity of microcephaly in patient 27.

Corpus callosum agenesis/hypoplasia and epilepsy are mostly associated with deletions of *ZBTB18* and *HNRNPU* genes in the patients with 1q43q44 deletion syndrome, respectively (34). However, none of these symptoms is demonstrated in the present case despite *ZBTB18* and *HNRNPU* disruptions. Recent studies have suggested that modifier genes or two-hit hypothesis may explain variable expressions among some patients with identical microdeletions. Another CNV is a disruptive singlebase- pair mutation in a related gene or an environmental event which influences the phenotype. These are all the examples of the second hit (35).

Patient 27 also had a duplication of about 541 Kb in Xp22.33 containing *PLCXD1, GTPBP6* and *PPP2R3B* genes as well as exons 1-3 and part of exon 4 of *SHOX* gene. *PPP2R3B* is one of the four major Ser/Thr phosphatases and negatively affects cell growth and division. *PPP2R3B* performs a regulatory control over the starting of DNA replication and its overexpression results in G1 phase cell cycle arrest (36).

There has been a variable impact on stature due to duplications of the region PAR1 containing the *SHOX* gene. Although some studies point to a correlation between *SHOX* gene deletions and short stature and Leri- Weill dyschondrosteosis, others have found some patients with the same clinical features who had partial or full duplications of *SHOX* gene. Bunyan et al. (37) reported a duplication of the exons 1-3 of the *SHOX* gene in a patient who had only short stature with no other clinical findings inherited from the proband’s affected mother. Co-overexpression of *PPP2R3B* and *SHOX* genes seems to have a synergistic effect on our proband’s short stature severity.

## Conclusion

In this study, 50 patients with MCA were studied in terms of chromosomal abnormalities and microdeletion/ microduplication syndromes. A patient was introduced with 46,XY, der(18)[12]/46,XY, der(18), +mar[8] dn as a novel case of MCA associated with hypogammaglobulinemia. Afterwards, given the highly complex and rare nature of the patient’s chromosomal abnormality, a novel mechanism was proposed to explicate the formation of both derivative and ring chromosome 18. Furthermore, a new 12q abnormality was found which had not been reported previously. In addition, a novel association of an Xp22.33 duplication with 1q43q44 deletion syndrome was demonstrated. The phenotypic analysis of the introduced patients can be useful for further phenotype-genotype correlation studies.
